# The National Dental Association, Division of the East

**Published:** 1898-05

**Authors:** 


### THE NATIONAL DENTAL ASSOCIATION, DIVISION OF THE EAST.

Upon another page our readers will find a call for a meeting at Albany, N. Y., to organize a branch of the National Dental Association for the Eastern States.

In our opinion this movement is not only premature, but it has not been called through the proper channels. No power exists in the organic law adopted at Old Point Comfort which gives to the officers of the national body the duty of organizing a branch, and this call is signed by the President and one of the Vice-Presidents of that organization. When the National Association has passed the ordeal of the first regular meeting it would then be appropriate to issue a call in proper order, but not until then.

The correct course would have been to send out a circular asking the co-operation of all subordinate associations requesting them to send delegates to an appointed place to confer as to the best means to take in organizing the proposed branch.

The course pursued has been the direct opposite of this. On the face of the call it appears to have had its origin in a self-constituted body, but as a matter of fact it was conceived in a single organization, and went no further, so far as society co-operation was concerned. The East has a large number of active organizations in which are to be found many of the most earnest workers in the dental profession, and this should have prevented arbitrary action without due consultation. The way the matter stands at present the ardor of many will be cooled and the work will be placed under the ban of suspicion, a most unfortunate result.

The dental profession is wearied with the methods of the past, and if we are to have simply a repetition of individual control, then it will be the duty of those having the best interests of the profession at heart to seek other avenues for the work of the future.

